# Prevention of mother-to-child transmission: experience of a Portuguese centre

**DOI:** 10.7448/IAS.17.4.19705

**Published:** 2014-11-02

**Authors:** Carmela Piñeiro, Sofia Fernandes, Cristóvão Figueiredo, Ana Sofia Santos, Marina Moucho, Rosário Serrão, Nuno Montenegro, António Sarmento

**Affiliations:** 1 Infectious Diseases Service, Centro Hospitalar São João, Porto, Portugal; 2 Obstetrics and Gynaecology Service, Centro Hospitalar São João, Porto, Portugal

## Abstract

**Introduction:**

HIV infection during pregnancy still raises controversial issues. Combined antiretroviral therapy (cART) has been successful in reducing mother-to-child transmission (MTCT). Routine screening in pregnancy and in pre-conception consultation proved to be one of the best methods able to get this treatment on time. We review our experience with pregnant patients with HIV infection.

**Materials and Methods:**

Retrospective and descriptive study. Data obtained from HIV-infected pregnant women from 1999 to 2012 with delivery and subsequent infectious diseases follow-up at our hospital.

**Results:**

We evaluated 136 patients (169 pregnancies), with a total of 147 living newborns (2 twin pregnancies) and 1 stillbirth. Median age at pregnancy was 30 (SD 5.7) years. Four patients were HIV-2 infected and one HIV-1+2 infected. 26 (19.1%) women were HCV co-infected and 6 (4.4%) HBV co-infected; 1 patient has HCV and HBV co-infection. Sexual risk for HIV acquisition was determined in 102 (75%) patients and 31 (22.8%) were intravenous drug users. 33/136 (24.2%) women were diagnosed on routine screening in pregnancy, 4 during delivery and 2 immediately after delivery. 36 (26.4%) patients had an AIDS-defining entity before pregnancy and no new opportunistic infections were diagnosed. ART was used in 157 (92.9%) pregnancies and 15 (9.5%) of them were treated only with NRTIs. At the time of delivery 86/144 (59.7%) patients had undetectable viral load (VL) (25 patients without VL determined), 91.7% of those on ART. 119 (70.4%) had a TCD4 cell count above 200 cells/mm^3^. MTCT occurred in 3/147 cases (2%): in one mother HIV-1 infection was diagnosed three weeks before delivery, other immediately after delivery and the third woman started cART (2NRTI+1PI/r) in the second trimester of pregnancy, always adherent and without secondary effects, VL at delivery was 50 copies/mL and elective C-section was performed.

**Conclusions:**

The fact that 24% of patients were diagnosed during pregnancy shows the importance of routine screening to all pregnant women. MTCT occurred in three children, but only one was administered cART for prevention.

